# A Review of the Robust Optimization Process and Advances with Monte Carlo in the Proton Therapy Management of Head and Neck Tumors

**DOI:** 10.14338/IJPT-20-00078.1

**Published:** 2021-06-25

**Authors:** Xiaodong Zhang

**Affiliations:** Department of Radiation Physics, The University of Texas MD Anderson Cancer Center, Houston, TX, USA

**Keywords:** robust optimization, Monte Carlo, adaptive planning, variable biological effect

## Abstract

In intensity-modulated proton therapy, robust optimization processes have been developed to manage uncertainties associated with (1) range, (2) setup, (3) anatomic changes, (4) dose calculation, and (5) biological effects. Here we review our experience using a robust optimization technique that directly incorporates range and setup uncertainties into the optimization process to manage those sources of uncertainty. We also review procedures for implementing adaptive planning to manage the anatomic uncertainties. Finally, we share some early experiences regarding the impact of uncertainties in dose calculation and biological effects, along with techniques to manage and potentially reduce these uncertainties.

## Introduction

Intensity-modulated proton therapy (IMPT) plans, when optimally designed, can exploit the advantages of proton beams to reduce the dose to healthy organs while delivering tumoricidal doses to the target volume. Given the dosimetric advantages of IMPT and complexity of anatomic targets and organs at risk (OARs) for head and neck (HN) cancer, most proton therapy centers treat patients with HN cancer with the pencil beam scanning technique [1]. IMPT techniques are more sensitive than other radiation therapy techniques to various uncertainties, which include (1) range uncertainty, (2) setup uncertainty, (3) anatomic change uncertainties, (4) dose calculation uncertainties, and (5) uncertainties associated with biological effects. Understanding the impacts of these uncertainties and compensating for them is the focus of treatment planning. The method developed to overcome range and setup uncertainties is called *robust optimization*, in which errors are incorporated directly into the optimization algorithm [2–6]; this approach is now used in clinical practice [2, 7, 8]. Two main techniques are used to manage anatomic change uncertainties: adaptive planning and multiple computed tomography (MCT) planning. Adaptive planning is considered the standard approach to manage the anatomic change uncertainties [9–11]. Some investigators have proposed that adaptive planning should be mandatory to ensure appropriate target coverage over the entire course of treatment [12]. Recently, it has been suggested that using MCT sets could reduce the number of adaptive plans necessary to address anatomic changes [13–15]. In IMPT planning, a correction factor of 1.1 is commonly applied to the absorbed dose because proton beams have a modestly higher radiobiological effectiveness (RBE) than photon beams. However, it is recognized that the RBE is variable over the depth dose curve; namely, it is higher in the region of the Bragg peak because of higher linear energy transfer (LET). The calculation of LET and variable RBE dose is often required in Monte Carlo (MC) dose calculation, and a new optimization strategy has been developed to optimize LET and RBE distribution [16–19].

This article is organized to provide a review of the robust optimization techniques developed to manage the abovementioned uncertainties. For the techniques available in commercial treatment planning systems (TPS), we focus on our clinical experience using those techniques. For the techniques that are under development, we share some recent results highlighting the need for the new techniques. Lastly, we provide our perspective on the future development of robust optimization and use of the MC algorithm in HN cancer.

## Review of Robust Optimization Technology to Manage Various Sources of Uncertainty

### Range and Setup Uncertainties

#### Beam-specific planning target volume for range uncertainties

**Figure 1.**
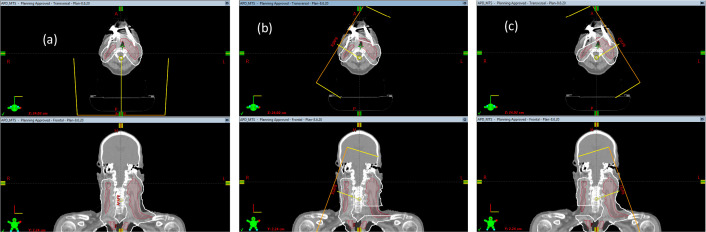
An example of bsPTVs in the clinical treatment planning process. The total target, which combines all CTVs, is shown in red. bsPTVs for posterior beam A (a), right lateral beam B (b), and left lateral beam C (c) are shown in white. bsPTVs for beam B and C not only include the distal and proximal margins but also avoid the metal artifacts. Also, part of the targets is cropped for bsPTVs of beam B and C, based on our experiences that beams B and C should not contribute to those cropped targets. Abbreviations: bsPTVs, beam-specific planning target volumes; CTV, clinical target volume.

The most important characteristic of protons with regard to their ability to spare normal tissues is the sharp drop-off in dose at the end of the particle range. However, because the translation of CT data from patient scans into Hounsfield units (HU) is at best an approximation, this process involves systematic uncertainties of about 3% in our understanding of exactly where protons stop in tissues (otherwise known as range uncertainties). The range uncertainties not seen in planning for proton, but not photon, therapies can be managed using the beam-specific planning target volume (bsPTV) approach [20]. The beam-specific target is the clinical target volume (CTV) or gross tumor volume, expanded laterally with margins similar to those used for the CTV-to-PTV margin but expanded distally and proximally with distal and proximal margins as determined by the range uncertainties. Currently, bsPTV can be created in the Eclipse TPS (Eclipse: Varian Medical Systems, Palo Alto, CA). If bsPTV is created for each beam, in principle, the treatment plan should be optimized by using the single field optimization approach. However, in real clinical practice, HN treatment plans often involve several targets and dose levels. And the complex geometric relationships between targets and OARs require multiple beams to contribute part of the target doses for optimal dose distributions for target coverage and OAR sparing. Multifield optimization, *where all spots from all fields were optimized simultaneously*, has to be adopted to create a plan with the best possible dose distributions. In practice, we often use the bsPTV to create a target for each beam to include the distal, proximal, and lateral margins. We also try to create the targets to avoid some anatomic regions, such as metal artifacts. We also intentionally avoid using beams from a particular direction to contribute to the dose to some portion of the targets. This process is often derived from our experience but is an important step in creating an optimal plan. Figure 1 shows 3 bsPTVs from 3 beam directions for a patient with HN cancer. These bsPTVs were created not for performing a single field optimization but for obtaining an optimal dose distribution in which doses were contributed from multiple beams.

#### Planning target volume in proton therapy for setup uncertainties

All radiation therapy modalities, regardless of whether they deliver protons or photons, involve uncertainties associated with setup that result from the misalignment of incident beams and patient anatomy and the realignment of internal heterogeneities among themselves and with respect to the target volume. For photon therapy, setup uncertainties are mitigated by the use of margins, specifically the margin obtained by expanding the CTV to the PTV. The margin approach works well for photon therapy because a photon dose distribution is relatively robust despite the presence of uncertainties. Unkelbach et al [5] described photon dose distribution as a “static dose cloud.” However, the margin approach does not work well for proton therapy. Distal and proximal to the beam direction, the margins for proton therapy should be determined by the range uncertainties. In the lateral direction, the static dose cloud will be broken under lateral setup uncertainties. *Also, the lateral penumbra is increasing when proton beam goes in depth*. The PTV is usually defined as the CTV plus a margin; one equation for determining that margin, based on the work of Van Herk et al [21, 22], is 2.5 Σ + 0.7 σ, where Σ is the systematic error and σ is the random error. If the PTV margin is set based on 2.5 Σ + 0.7 σ, then the CTV has a 95% chance of receiving the prescribed dose (based on the treating facility's systematic and random error) if the prescribed dose to the PTV is 95%. This margin formula has been widely adopted as a guideline for evaluating the robustness of photon therapy. However, the use of PTV is increasingly understood to be inappropriate for evaluating the robustness of proton plans, especially IMPT [23]. To deal with the range and setup uncertainties, all TPS have implemented a robust optimization approach, in which errors are incorporated directly into the optimization algorithm; this approach is now used in clinical practice. Robust optimization can be done in several ways, among them the probabilistic approach and worst-case optimization. The probabilistic approach [5, 6] assumes prior knowledge of the uncertainty's probability distribution, which is assumed to be normal in most cases. Worst-case optimization, on the other hand, seeks to optimize the worst case that could occur. Pflugfelder et al [4] implemented the first version of worst-case optimization in their in-house particle therapy TPS (KonRad, Heidelberg, Germany). Worst-case dose optimization, with slight variations, has now become the in-house standard at MD Anderson and commercially [3, 8, 24, 25]. The worst-case optimization was first formulated [3] as:

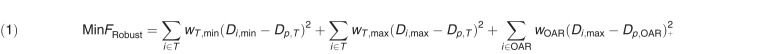



The terms *D_i_*_,min_ = 
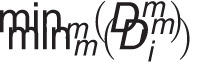
 and *D_i_*_,max_ = 
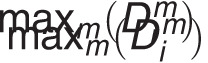
 indicate the minimum and maximum dose among *m* possible doses 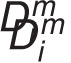 in voxel *i*, which are calculated as 
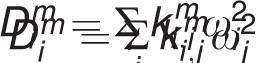
 for each iteration. The *m*th influence matrix 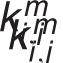, incorporating either range or setup uncertainties, is precalculated before optimization and stored in the TPS memory. Notably, the optimization target *T* is the CTV rather than the PTV in Equation 1. Also, each dose is forced to use either minimum or maximum dose among *m* possible doses. We refer to this kind of objective as *robust objectives*. In clinical practice, it may be necessary to compute selected objectives by using only the nominal dose distribution in robust optimization; such objectives can be referred to as *nominal objectives*. We call this approach to robust planning, in which robust objectives and nominal objectives are selectively applied on different planning structures, *selective robust optimization*; this approach has been implemented in collaboration with Eclipse into their TPS.

In our practice, we use 8 different uncertainty scenarios and plot the banded dose-volume histogram (DVH) with a total of 9 scenarios (the 8 perturbed conditions + the original one). The 8 scenarios are calculated by shifting the isocenter of the original plan by +dx, +dy, +dz and overshooting/undershooting the range by +dr, where dx, dy, and dz are the systematic uncertainties in the x, y, and z directions and dr is the range uncertainty (typically assumed to be 3.5%). We used an approximation that assumes that 95% coverage of the worst-case CTV would be equivalent to 95% coverage of the PTV in photon plans. This concept is an approximation and has not been rigorously proved to be valid. The assumptions underlying the 9-scenario approach may not be valid for the following reasons. First, it is not always appropriate to assume that only cold spots have negative effects in treating the target. Second, the error on a perturbation dimension, say dx, cannot be +3 mm and –3 mm at the same time. Third, worst-case analysis examines the shift in x, y, and z directions and beam range uncertainties separately, but shifts always happen in all directions simultaneously with range uncertainties, making it unclear if this type of analysis would overestimate or underestimate the sensitivity of a plan to uncertainties. Recently, we did a study [26] to test the hypothesis that the worst-case robust optimization strategies could cover most possible setup and range uncertainties in real scenarios. We performed a comprehensive simulation in which the dose for each plan was recalculated 625 times by using Gaussian systematic setup and proton range uncertainties for 7 patients with HN cancer treated in our center. Subsequently, from the simulation results, we calculated the target coverage in all perturbation scenarios as well as the ratios of target coverage located within the threshold of 8 worst-case scenarios. We found that the probability that the perturbed-dose indices of the CTVs in each scenario were within the worst-case scenario limits ranged from 89.51% to 91.22% for both the nominal and worst-case robust optimization IMPT plans. For this study, we concluded that the worst-case strategy for robust optimization adequately models and covers most of the setup and range uncertainties for the IMPT treatment of the patients with HN cancer [26].

#### Anatomic change uncertainties

During radiation therapy, changes in patient anatomy can take place that themselves introduce uncertainties. For instance, throughout the course of treatment for HN cancer, patients may experience severe interfractional anatomic changes, including shrinkage of primary tumors or nodal lesions, resolution of postoperative changes or edema, changes in nasal cavity filling, and weight loss [27–29]. We recently reported examples of these anatomic changes in patients with HN cancer during radiation therapy.

#### Adaptive planning

**Figure 2.**
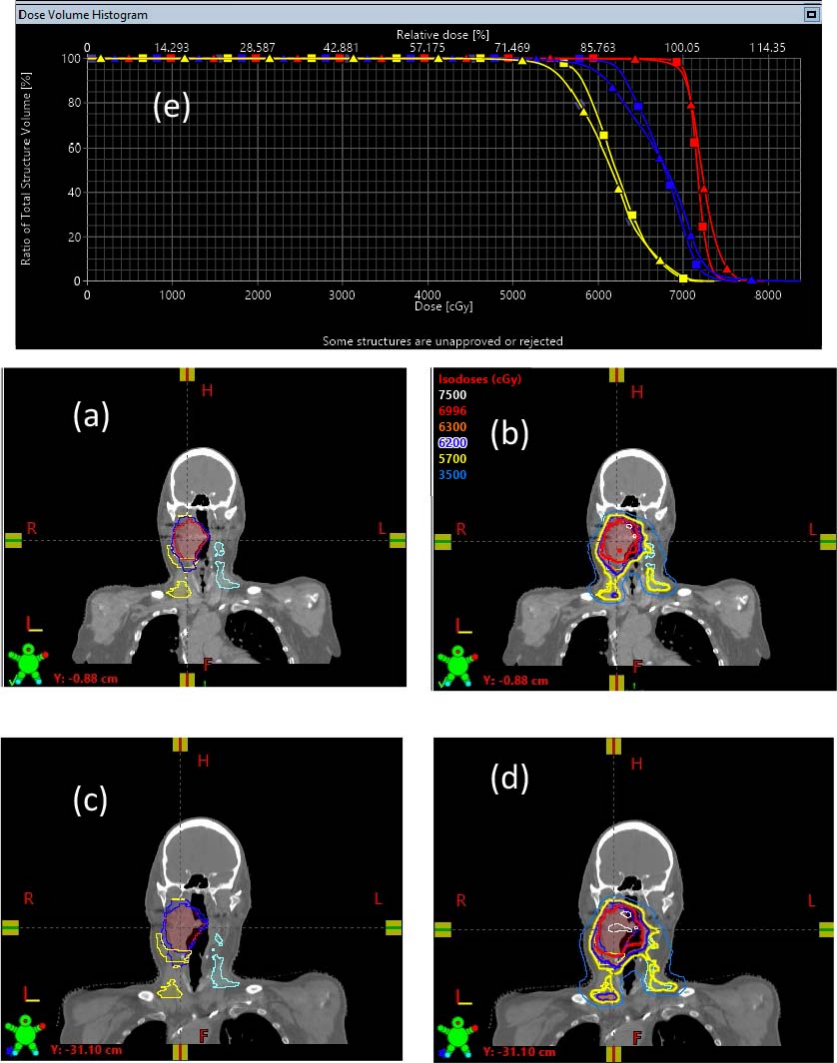
An example of verification planning process that triggered adaptive planning. (a and c) CT images and target contours (red: CTV 6996; blue: CTV 6300; yellow CTV 5700 [right]; cyan: CTV 5700 [left]) at planning (a) and verification (c). Target shrinkage can be seen by comparing (a) and (c). (b and d) Dose distributions using CT (a) and (c). (e) Dose-volume histograms at planning (square) and verification (triangle). Abbreviations: CT, computed tomography; CTV, clinical target volume.

The main method to manage the anatomic uncertainties is adaptive planning [9, 10]. Some investigators have proposed that adaptive planning should be mandatory to ensure appropriate target coverage over the entire course of treatment. For HN cancer, our previous experience indicates that significant deviations in body contours due to weight loss begin about 4 weeks after the start of treatment [30, 31]. On the basis of those findings, patients receive radiation to bilateral neck volumes, and verification CT plans are created mandatory before treatment and at 4 weeks (± 1 business day) after the beginning of treatment. In our practice, almost all patients have verification plans and more than 90% have adaptive plans (re-planning was necessary in some cases before 4 weeks owing to poor initial setup). In the verification planning process, the verification CT images are registered with the original planning CT images by using a commercially available deformable image registration tool (eg, Velocity, Varian Medical Systems, Palo Alto, CA). The treatment targets and the major OARs, such as the parotids, oral cavity, spinal cord, and brainstem, are deformed and transferred from the original CT images to the verification CT image data set. All deformed contours are reviewed by a radiation oncologist before the adaptive plan is generated. The beam configuration and intensity distribution profile from the original treatment plan are then copied to the verification CT image set to create a verification plan, from which updated radiation dose levels reflecting the most recent anatomic changes are calculated. With the dosimetric results, the radiation oncologist then evaluates and compares the scan sets to determine whether adaptive treatment plans are needed. Figure 2 shows a verification planning process that triggered adaptive planning to compensate for a dosimetric change due to tumor shrinkage.

**Figure 3.**
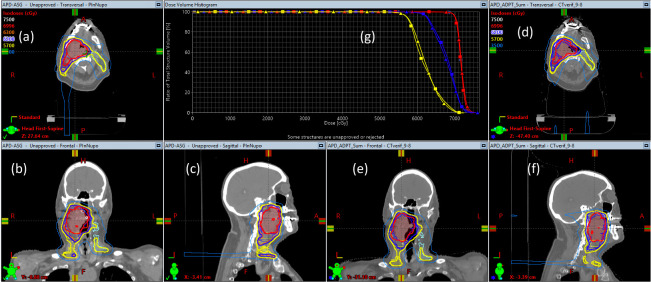
An example of adaptive planning restoring the planned dose distribution. (a-c) Dose distribution of the original plan. (d-f) Dose distribution of the adaptive plan. The dose distribution in (d), (e), and (f) is the summation of the original plan for 23 fractions and adaptive plan for 10 fractions showing on the CT at verification. (g) Dose-volume histograms for the original plan (square) and summation of the original plan and adaptive plan (triangle). Abbreviation: CT, computed tomography.

For adaptive planning, no significant dosimetric differences should be present between the original and adaptive plans because the re-planning process should mimic and reproduce the original planning goals with the specified dose constraints. As shown in Figure 3, the cumulative dose from the summation of the original plan for 23 fractions and adaptive plan for 10 fractions indeed restored the dose distribution in the original planning process.

#### MCT optimization

However, the adaptive planning process increases the workload for physicians and physicists and increases the economic burden for patients. In our center, adaptive planning for patients with HN cancer represents one of the most time-consuming clinical workflows. The workload for each adaptive plan is almost equivalent to that of a new plan for a new patient. Given the need to improve clinical efficiency, the MCT optimization technique was recently proposed for the reduction of adaptive planning in IMPT for HN cancers. The MCT optimization technique involves 2 CT data sets: the primary planning CT (PCT) and the first adaptive planning CT (ACT1) scans. The optimization includes the primary structures of the PCT and adaptive structures of the ACT1. In our in-house optimization system [3, 8], we used a standard quadratic objective function, and each iteration of the objective function of MCT was obtained by adding together the objective functions of the PCT and ACT1. To facilitate MCT optimization, ACT1 images were first rigidly registered to the PCT images, and then the plan isocenter of ACT1 was calculated according to the registration results. In our recent study, we retrospectively selected 10 patients who went through at least 2 adaptive planning processes. We used the second adaptive CT (ACT2) to validate that MCT optimization. We found that MCT plans for all patients met all target dose and OAR criteria for all 3 CT data sets. This result indicated no second adaptive plan would be needed if MCT optimization techniques had been used in the first adaptive planning process. The drawback of MCT optimization is that this technique requires a second CT data set (ACT1) with relatively large anatomic change. Some researchers have begun using artificial intelligence approaches to predict the anatomic change, and this prediction can be used to generate a synthetic CT. This approach is promising. As we accumulate enough data, we might reduce the need for adaptive planning and significantly improve our ability to manage anatomic change uncertainty.

#### Dose calculation uncertainties

In clinical practice, the analytic pencil beam (PB) algorithm is commonly used in IMPT dose calculation because of its accuracy and fast calculation speed. However, the validated MC algorithm is generally considered the most accurate method in dose calculation and is becoming available in commercial TPS. Between the PB and MC algorithms, the dosimetric indices of the targets *and critical structures such as brainstem, spinal cord, and optical pathways* agreed within 4% in recent reports [32, 33].

**Figure 4.**
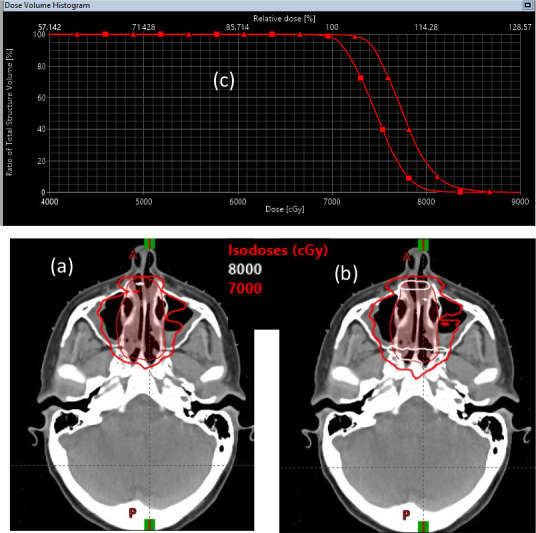
An example of the different dose distributions calculated with the PB algorithm and MC algorithm. The dose distributions calculated with PB (a) and MC (b) for a 2-beam plan are shown. (c) Dose-volume histograms of clinical target volume 70, calculated with PB (square) and MC (triangle) algorithms. Abbreviations: MC, Monte Carlo; PB, pencil beam.

**Figure 5.**
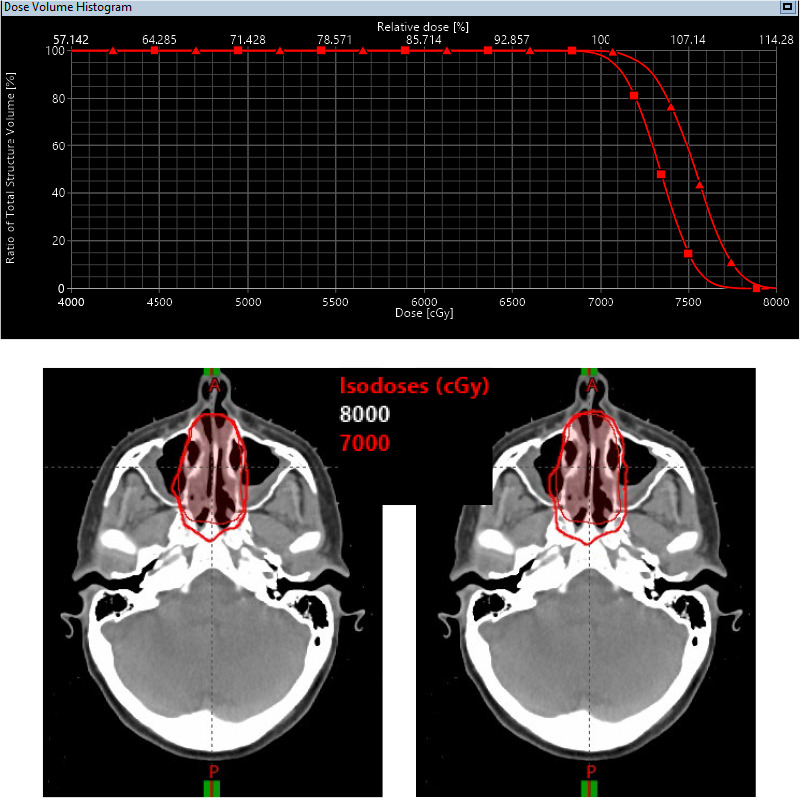
An example of the different dose distributions calculated with the PB algorithm and MC algorithm for a plan with 8 beams. The dose distribution calculated with PB (a) and MC (b) for an 8-beam plan is shown. (c) Dose-volume histograms of clinical target volume 70, calculated with PB (square) and MC (triangle). Abbreviations: MC, Monte Carlo; PB, pencil beam.

The difference between PB and MC dose calculation algorithms has 2 major impacts. The first is the dose calculation uncertainties between the PB and MC algorithms. Figure 4 shows the dose distributions calculated by PB algorithm and MC algorithm for a plan using 2 lateral beams. This was an extreme case in which the target was in a heterogeneous anatomic region where we observed the largest discrepancy between MC and PB algorithms. In this scenario, the mean doses to the target (CTV70) were 74.7 Gy and 77.7 Gy and the maximum doses were 86.8 Gy and 95.1 Gy, respectively, between PB and MC algorithms. The discrepancy between the mean dose was about 4%, but the discrepancy between the maximum dose was about 8%. In the literature, dose discrepancies exceeding 4% were rarely reported in clinical patient treatment plans. This observation is illustrated by Figure 5, in which mean doses of CTV70 were 73.3 Gy and 75.3 Gy and the maximum doses were 79.6 Gy and 81.8 Gy, respectively, between the PB algorithm and MC algorithm for an 8-beam plan. The discrepancy is below 3% for both the mean and maximum doses. These examples have some important implications for treatment plan design and dose calculation algorithms. More beams are desired to create optimal dose distributions IMPT planning. It was previously conceived that proton plans can be designed by using fewer beams than IMRT plans since the depth direction of proton plans can be modulated. However, for an extreme case such as the one shown in Figure 4, it was impossible to achieve a conformal isodose line to the target with 2 beams. Even with 8 beams, we have not achieved perfect conformality. This fact led the proton community to explore the proton arc therapy. As shown in Figure 5, multiple beams also have an effect of reducing the discrepancy between PB and MC dose calculation algorithms.

The second impact of discrepancy between the dose calculation algorithms is the convergence error. Recently, we observed that treatment plans optimized by using the PB algorithm actually did not reach the optimal solution evaluated by using the MC dose calculation algorithm, especially for IMPT treatment planning considering the effect of LET/RBE. For LET/RBE-based IMPT planning design, we strongly recommend using the MC dose calculation algorithm not only for the plan evaluation but also for the optimization process.

#### Biological uncertainties

**Figure 6.**
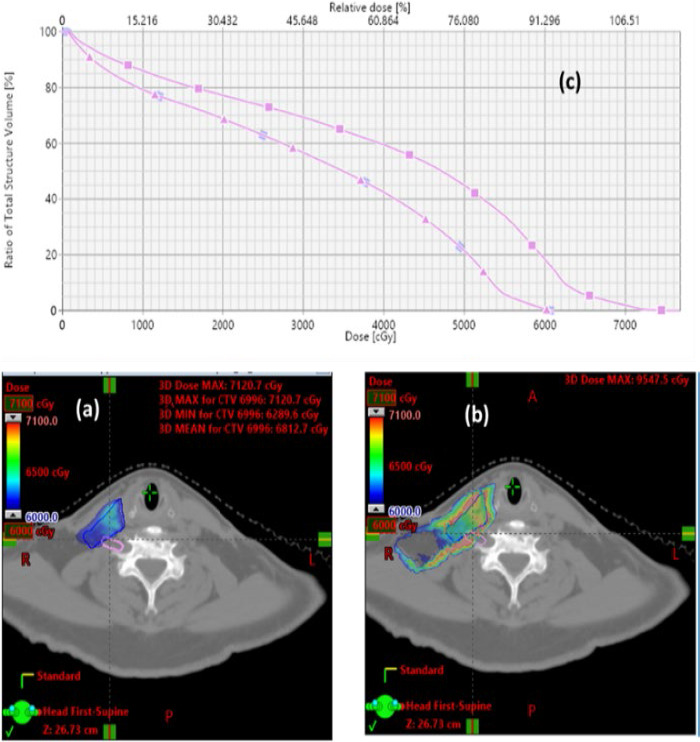
Dose distribution with constant RBE of 1.1 (a) and variable RBE model (b) in the right brachial plexus region of a patient with head and neck cancer. Dose-volume histograms (c) of the right brachial plexus with constant RBE (triangle) and variable RBE (square) are shown. Irradiation of the right brachial plexus can cause permanent arm damage. Abbreviation: RBE, radiobiological effectiveness.

Currently, most proton therapy centers use constant RBE dose to evaluate the dose distribution. However, more and more evidence suggests that variable RBE calculation should be performed for patients undergoing proton therapy. Some higher-than-expected toxic effects such as brain necrosis [34, 35] and skin toxicities [36] are possibly related to higher-than-expected RBE doses in some anatomic regions. Figure 6 displays dose distributions with constant RBE of 1.1 and variable RBE model in the right brachial plexus region in a patient who developed arm injury after proton treatment in our center. Although no variable RBE model has been validated to evaluate the treatment plan in clinical setting, at MD Anderson, more and more clinicians have started using the variable RBE model [37] as a reference when evaluating a proton therapy plan.

The variable RBE dose is a function of LET, *dose fractions*, and the alpha/beta ratio of the tissues. LET is a physical quantity that does not depend on the biological properties of the tissues. As a surrogate of treatment planning optimization based on variable RBE dose, an optimization was proposed that optimized the physical dose and LET simultaneously. Two plans might have the same physical dose distribution but different LET distributions. Ideally, an IMPT plan should have high LETs in target volumes and low LETs in critical structures and normal tissues. Cao et al [17] added LET-based objectives for maximizing LET in target volumes and minimizing LET in critical structures and normal tissues. They compared LET-incorporated optimization and the conventional dose-based optimization for 5 cases of proton therapy to brain tumors. They found that the 2 optimization approaches generated comparable physical dose distributions. However, the LET-incorporated optimization achieved lower LET values in critical structures, such as the brainstem and optic chiasm, and higher LET values in target volumes than did the dose-based optimization. They concluded that the inclusion of LET-dependent criteria in IMPT optimization could lead to similar dose distributions as the conventional optimization but superior LET distributions in target volumes and normal tissues.

The LET-incorporated optimization approach proposed by Cao et al [17] has been adopted to manage biological uncertainties in some clinical trials for brain and anal cancer in our institution. A variation of this approach using track-based objectives has been implemented in the RayStation TPS (RayStation: RaySearch Laboratories, Stockholm, Sweden) [18]. But most centers do not have enough clinical experience to adopt this approach, and how much biological uncertainty can be mitigated by this approach is unknown.

#### Conclusions and outlooks

Thanks to efforts in basic algorithm development and translation research, the robust optimization approach to manage range and setup uncertainties is becoming the conventional technique for clinical treatment planning. Furthermore, anatomic change uncertainties are well managed by adaptive planning. However, similar to the development of robust optimization to manage range and setup uncertainties, basic algorithm development has shown that anatomic change uncertainties can be better managed by MCT optimization and biological uncertainties can potentially be managed by incorporating LET/RBE into the optimization process. MCT optimization and LET/RBE-based optimization have not been routinely adopted but are expected to be adopted routinely since some TPS have begun providing those capabilities. However, we feel uncertainties can be better managed with the following future improvements:

1. Better tools to evaluate and compare uncertainties: Trofimov et al [38] reported that the histograms from various scenarios were combined to create DVH bands to illustrate possible deviations from the nominal plan for the expected magnitude of setup and range errors. The DVH band method has been used in many studies to evaluate robustness but has not been implemented in any commercial TPS. Also, it would be useful to compare 2 DVH bands side by side to compare the robustness of the 2 plans. *This effort is complicated since the correlation of local control and robustness needs to consider the compounding factors such as biological tumor characteristics and combinations of antineoplastic drugs*.
2. Criteria for the robustness: A robust plan comes with a cost. Too much robustness will scarify some normal tissue. Currently, in the clinical scenario, there are no standard criteria to evaluate the robustness. An effort is under way at our institution to define the robustness with clinical outcome data such as the local control data extracted from the patients with HN cancer treated by IMPT.
3. On-line adaptive planning: The best way to manage setup and anatomic change is to design the treatment plan while the patient is still on the treatment couch. This on-line adaptive technique is slowly becoming available in photon therapy owing to the MRI-Linac. With better and better on-board imaging mounted on proton gantry, we expect that on-line adaptive planning will come to proton therapy.
4. Manage biological uncertainties by using better delivery techniques: Although the LET/RBE-based optimization technique has been developed, the ability to manage biological uncertainties by current delivery techniques, which normally use 2 to 5 beams, is very limited. We already demonstrated that more beams will create better plans and reduce dose calculation uncertainty. We expect that LET/RBE uncertainty will be better managed by using more beams and potentially proton arc therapy.
